# Capturing Eating Behavior from Video Analysis: A Systematic Review

**DOI:** 10.3390/nu14224847

**Published:** 2022-11-16

**Authors:** Michele Tufano, Marlou Lasschuijt, Aneesh Chauhan, Edith J. M. Feskens, Guido Camps

**Affiliations:** 1 Division of Human Nutrition and Health, Wageningen University & Research, Stippeneng 4, 6708 WE Wageningen, The Netherlands; 2 Wageningen Food and Biobased Research, Wageningen University & Research, Stippeneng 4, 6708 WE Wageningen, The Netherlands; 3 OnePlanet Research Center, Plus Ultra II, Bronland 10, 6708 WE Wageningen, The Netherlands

**Keywords:** eating behavior, computer vision, AI, automatic analysis, healthy eating

## Abstract

Current methods to detect eating behavior events (i.e., bites, chews, and swallows) lack objective measurements, standard procedures, and automation. The video recordings of eating episodes provide a non-invasive and scalable source for automation. Here, we reviewed the current methods to automatically detect eating behavior events from video recordings. According to PRISMA guidelines, publications from 2010–2021 in PubMed, Scopus, ScienceDirect, and Google Scholar were screened through title and abstract, leading to the identification of 277 publications. We screened the full text of 52 publications and included 13 for analysis. We classified the methods in five distinct categories based on their similarities and analyzed their accuracy. Facial landmarks can count bites, chews, and food liking automatically (accuracy: 90%, 60%, 25%). Deep neural networks can detect bites and gesture intake (accuracy: 91%, 86%). The active appearance model can detect chewing (accuracy: 93%), and optical flow can count chews (accuracy: 88%). Video fluoroscopy can track swallows but is currently not suitable beyond clinical settings. The optimal method for automated counts of bites and chews is facial landmarks, although further improvements are required. Future methods should accurately predict bites, chews, and swallows using inexpensive hardware and limited computational capacity. Automatic eating behavior analysis will allow the study of eating behavior and real-time interventions to promote healthy eating behaviors.

## 1. Introduction

Eating behavior determines the nutritional intake and the health status of adults and children. Eating behavior is defined as the ensemble of food choices, eating habits, and eating events (bites, chews, and swallows) [1]. Eating rate, which is the amount of food consumed per unit of time (g/min), can affect food intake [2], energy intake [3], and weight gain [4,5], as well as the risk of obesity [6,7], and metabolic diseases [8,9]. Eating behavior can be influenced by the food consumed, although it develops through parent–child interactions, individual child growth, neural mechanisms, and social influences [10]. For example, eating rate is an individual trait but it strongly depends on food properties, such as food texture and matrix [11,12]. Solid foods with hard textures (difficult to bite and chew) decrease eating rate, food intake, and energy intake, whereas semi-solid or liquid foods increase eating rate, food intake, and energy intake [13]. To prevent food overconsumption and obesity, interventions in food texture and eating rate can manipulate individual eating behavior and lower food and energy intake [14,15].

Tracking each eating episode (i.e., a meal) is crucial for a valid comprehension of individual eating behavior. The golden standard for this process consists of two or three independent researchers that watch the videos of each eating episode and record the eating behavior events [16]. For the annotation, the number of eating events, bite-size, chewing frequency, eating rates, meal duration, and rate of ingestion [17] must be recorded. Measuring eating behavior events requires the training of human annotators and often the purchase of expensive software licenses. The most used software packages for eating behavior annotation are Noldus Observer XT (Noldus, Wageningen, the Netherlands) [18], ELAN 4.9.1 Max Planck Institute for Psycholinguistics [19], and ChronoViz [20]. Although human annotation can be accurate, often this task is prone to subjectivity and attentional lapses, due to its repetitive and time-consuming nature. Furthermore, large prospective studies are unfeasible due to the large number of videos to annotate. Because of this, the evidence in the eating behavior field is confined to cross-sectional and short-term experimental studies [3,21]. Therefore, according to the experts in the field, the human annotation process should be automated [22,23].

Despite the recent advancements in smart devices for tracking eating behavior, including the wristband [24], ear sensors [25], smart fork [26], smart utensils [27], smart plate [28], smart tray [29], and wearable cameras [30], the video recordings of eating episodes remain the least intrusive and most scalable approach. Video recordings are able not only to reproduce wearables functionalities (e.g., eating rate, number of bites) but also to expand them towards more complex eating behavior events (e.g., emotion detection for eating behavior, social interactions at the table, or parent–child interaction [31]).

Such fortes make video recordings of eating episodes a strong candidate for tracking eating behavior. The automatic analysis of video recordings can replace the expensive and time-consuming manual annotation and lead to better interventions to manipulate eating behavior. However, it remains unclear what methods are applicable to analyze meal videos automatically.

Therefore, the aim is to determine accuracy, advantages and disadvantages of the current video-based automated measures of eating behavior. This review focuses on video-based methods that aim to predict bites, chews, swallows of consuming foods and food liking.

## 2. Materials and Methods

### 2.1. Search Strategy

This systematic review was performed to assess the available methods to automatically detect eating behavior events. The Preferred Reporting Items for Systematic Reviews and Meta-Analyses (PRISMA) guidelines were followed for the literature search [32]. The databases PubMed, Scopus (Elsevier), ScienceDirect, and Google Scholar were screened. The terms included in the search strategy were eating, behavior (or behaviour), video, methodology and further analysis terms (see Appendix A for the comprehensive list of search queries). Additional author search was performed for the most common authors found, using the snowballing search strategy. All the citations were exported to the reference manager software Zotero (version 5.0.96.3), where the first author (MT) screened all the titles and abstracts to select the scientific publications that met the criteria as outlined below.

### 2.2. Inclusion and Exclusion Criteria

Original research articles were considered as valid exclusively if published in the English language and containing findings on video analysis for human eating behavior from January 2010 to December 2021. This temporal cut-off was chosen to ensure that outdated (computer vision and machine learning) technologies would be excluded. Conference papers were included. These studies might contain preliminary results therefore validity and precision in Table 1 were considered while writing this review. Articles concerning non-human studies were excluded. We excluded research articles on eating behavior with video electroencephalogram monitoring, verbal interaction analysis, or sensors, as well as research studies not focusing on automated measures as they are beyond the scope of video analysis.

### 2.3. Article Selection

A complete overview of the selection process is depicted in the PRISMA flow diagram [32] (See Appendix A). 239 records were found through database searching and 38 through author search, for a total of 277 records identified from all sources. After removing 20 internal or external duplicates, 257 unique research articles were assessed. To determine their eligibility, the research articles were screened based on their titles and abstracts. The main exclusion criterium was the absence of video analysis from the title and abstract (n = 92). We discarded the publications not concerning eating behavior research (n = 57). These publications reported about types of behavior not related to eating behavior and were therefore unrelated. Non-human studies (n = 23), non-original research studies (n = 26), and publications written in a language other than English (n = 7) were considered non-eligible. The full text of each of the remaining scientific publications (n = 52) was screened rigorously. The articles were excluded due to the following criteria: videos on EEG monitoring (n = 2), eating behavior not including automated measures(n = 15), using verbal interactions between patients and caregivers for eating behavior (n = 6), published before 2010 (n = 6), and using sensors or wearables for tracking eating behavior (n = 10). Finally, the remaining 13 publications were included in this study for review and data extraction.

### 2.4. Summary Characteristics and Data Extraction

For all the eligible studies, study characteristics such as the methodology used, the research outcomes, and the validation procedure were retrieved. Additionally, the precision of the outcomes was reported using the metrics from the scientific publications: F1-score, accuracy score, precision score, or average error with standard deviation. We tabulated the results from the original publications and summarized the data narratively. The methods were classified by the authors based on their similarities and dissimilarities. This resulted in 5 categories: facial landmarks, deep learning, optical flow, active appearance model, and video fluoroscopy. The extraction was performed by one author (MT). Since this review is centered on the methodology rather than on the outcomes of the studies, the risk of bias was not assessed.

## 3. Results

The literature was reviewed systematically according to the PRISMA guidelines to assess the methods to automatically detect eating behavior from video recordings. Overall, the main methods found were facial landmarks, deep learning, optical flow, and active appearance model which can be combined to detect bites, chews, food intake, and food liking. Video fluoroscopy was the only method applied to detect swallows. Facial landmarks are the most used method for detecting bites and chews automatically from video recordings of eating episodes. A summary of the main methods is provided in the next section with their application for eating behavior events (Table 1).

### 3.1. Facial Landmarks

Face detection is a computer technology able to recognize human faces in an image or video. Facial landmarks (or key points) detection is a computer vision task to localize and track key points on a human face.

Several open-source computer vision packages have been developed for facial recognition and landmarks. OpenSMILE is a toolkit for facial detection, which can also extract and analyze audio features for sound and speech interpretation [46]. OpenFace is an open-source package for facial landmark detection, eye-gaze and head pose estimation with real-time performance (using a common webcam) [47]. OpenPose is an open-source package for multi-person, real-time, 2D pose detection with facial, body, hand, and foot landmarks [48]. The Viola-Jones face detector is a machine learning framework for face detection. It combines Haar-like Features, the AdaBoost Algorithm, and the Cascade Classifier, and it can also be adapted to object detection [49]. The Kazemi and Sullivan landmark detector is a machine learning framework for face alignment. It uses an ensemble of regression trees to estimate the facial landmarks from the pixels of an image or video [50].

Facial landmarks (or detection) were used in 7 out of 13 (54%) of the studies included. To predict food liking, Hantke et al. (2018) applied OpenFace for facial landmark extractionduring the 2018 EAT challenge. The participants scored their food preferences using a continuous slider with values between 0 (extremely dislike) and 1 (extremely like), which were later mapped into two variables (‘Neutral’ or ‘Like’) using a threshold. Facial landmarks were used to predict food liking. The mouth-related subset of landmarks yielded a better performance than landmarks from the entire face, which showed overfitting. A support vector machine (SVM) was optimized using Leave-One-Out cross validation to recognize food liking automatically (accuracy: 0.583) [35].

To classify food liking, Haider et al. (2018) used the OpenSMILE [46] package for facial landmarks coupled with audio feature extraction. In a Leave-One-Out cross validation setting, they employed the active feature transformation method to find a subset of 104 features that provided better results (Unweighted Average Recall = 0.61) for food liking (out of the 988 features from the whole dataset) [36].

To detect eating activity, Nour et al. (2021) [42] used 68 facial landmarks to locate the mouth and OpenPose to detect hands during eating episodes [48,51]. Eating activity was detected when the hands were in proximity of the mouth (accuracy: not reported) [42].

To detect chewing, Alshboul et al. (2021) used the Viola-Jones face detector [44] to detect faces and the Kazemi and Sullivan landmark detector [50] to apply facial landmarks. The videos were recorded outdoors, indoors, and in public spaces, with different light intensities. The video frame rate was 30 fps. The Euclidean distances were calculated between the jaw and mouth landmarks and a reference point (upper left corner of the face rectangle) [44].

To automatically detect bites, Kostantinidis et al. (2019) extracted x- and y- mouth features coordinates applying OpenPose in videos of eating episodes. Subsequently, the coordinates were used to model bites, by calculating the mouth corner between mid-upper and mid-lower lips (the classification task was performed with deep neural networks, see Section 3.2) [37].

To estimate food intake, Okamoto et al. (2014) restricted the detection area of the Viola-Jones face detector to the lower half of the face to minimize wrong predictions [34]. Interestingly, Okamoto et al. (2014) developed a chopstick detector by separating the front portion of the image from the background and applying the OpenCV Hough transform [52] for straight-line detection to enhance linear object detection (i.e., chopsticks) [34].

To feed people with impaired mobility, Park et al. (2020) developed a robotic system with facial landmarks. The system localizes the user’s face and detects 68 facial landmarks using dlib [51] with the histogram of oriented gradient feature, a sliding window detector, and a linear classifier. They improved the model for light variations, 3D facial estimation, and facial orientation to detect when the mouth is opened. The facial recognition system is combined with a mobile manipulator to automatically deliver the food to the user’s mouth [43]. In conclusion, facial landmarks can predict bites and chews, although the camera angle can impact their performance and only 2D facial landmarks have been tested so far.

### 3.2. Deep Neural Networks

Deep learning approaches are a subset of machine learning methods, in particular artificial neural networks, designed to automatically extract representations (also known as features) directly from raw input [53]. In deep neural networks, as the network gets deeper, several levels of features (from raw input to more and more abstract representations) are extracted by composing simple non-linear modules (artificial neurons distributed over hidden layers). Unlike conventional machine learning methods, these features are not usually designed by humans, and learned directly from raw data using a general-purpose learning procedure.

A multitude of deep neural networks have been proposed in literature which are distinct in design and architecture [53,54,55].

Numerous deep neural networks are commonly used for image and video classification tasks. Convolutional Neural Networks (CNNs) are an example of deep neural networks. CNN is an architecture inspired by biological neuron connections. It consists of an input layer, hidden layer(s) and an output layer which are connected by activation functions. Some CNNs can be differentiated based on the input file. 2D-CNNs are commonly used to process RGB images (or video frame by frame). In contrast, 3D-CNNs use a tridimensional input file, such as a video file or a sequence of 2D frames. Other CNNs can combine different input files. For example, Two-Stream CNNs combine data from the RGB-images with optical flow for action recognition.

CNNs can analyze input with a temporal component. CNN-LSTM (Long Short-Term Memory) with feedback connections are well suited for time-series data. SlowFast combines a slow and fast pathway for analyzing the dynamic and static content of a video.

Some CNNs are specialized in object detection (e.g., Faster R-CNN [56]) and instance segmentation (e.g., Mask R-CNN [57]). To detect an object, Faster R-CNN replaced the selective region search with a region proposal network, which boosts the detection task. Mask R-CNN are an extension of Faster R-CNN. After the region proposal network, Mask R-CNN classifies the region and then it classifies the pixels within the region to generate an object mask. CNNs were used in 4 out of the 13 (30%) of the included studies, which assessed CNNs performance for a given classification task (e.g., bite or no-bite).

To assess food intake in shared eating settings, Qui et al. (2019) rescaled the videos from a 360-degree camera to use it as the input for Mask R-CNN [38]. In this free-living setup, a 360 camera recorded a video from the center of a table where three subjects shared a meal. For each subject, a box was applied on the food, person, face, and hands. When the distance among the pixels in the face-hand-food boxes was relatively short, the system predicted a dietary intake event (accuracy not reported) [38]. To detect a bite, Rouast et al. (2020) investigated 2D-CNN (F1-score: 0.795), 3D-CNN (F1-score: 0.840), CNN-LSTM (F1-score: 0.856), Two-Stream CNN (F1-score: 0.836), SlowFast (F1-score: 0.858). The SlowFast with ResNet-50 architecture is the best model to predict a bite through intake gesture detection. The video sessions were recorded using a 360 camera on the table where four subjects shared a meal [40].

Kostantinidis et al. (2020) combined facial landmarks (OpenFace) with CNNs [41] to develop RABiD, a deep learning-based algorithm, for bite classification. RABiD combines temporal and spatial interactions (convolutional layers, max-pooling steps, LSTM, and fully connected layers) in a two-data stream deep learning-based algorithm [41]. In RABiD, the first data stream uses 2D features from mouth corners, while the second data stream uses 2D features from the upper body. The mouth, head, and hands predicted bites with F1-score of 0.948 [41].

To count bites automatically, Hossain et al. (2020) used a Faster R-CNN. Initially, human raters manually marked the participants’ faces as the region of interest to train the Faster R-CNN for face detection. The bite images consisted of image frames including the face together with straw/glass/bottle/hand/spoon/fork and food in the field of view. A binary image classifier (with AlexNet architecture) was trained to distinguish between ‘bite’ images and ‘non-bite’ images. This method achieved an accuracy of 85.4% ± 6.2% in counting bites automatically in 84 videos of eating episodes [39]. In summary, deep neural networks can detect human body and predict bites. However, deep learning is not efficient in predicting chewing and it requires expensive hardware and software requirements.

### 3.3. Optical Flow

Optical flow is a computer vision approach that tracks motion of surfaces, objects, and edges between consecutive image frames. Each image frame is converted to a 3D vector field to describe space and time. The spatial motion is calculated on the 3D vector fields at every pixel [58]. The resulting values (or parameters) can be used to assess the movement of any object using videos as input.

Optical flow was used in 2 of the 13 (15%) included studies. To estimate chewing activity, Hossain et al. (2020) used optical flow to extract spatial motion parameters from the jaw (accuracy: 88.64% ± 5.29% in 84 meal videos) [39]. To detect a bite, Rouast et al. (2020) used a motion stream with the horizontal and vertical components of the optical flow. The motion stream was integrated into a 2D-CNN. The models using optical flows (Small 2D-CNN, F1-score: 0.487; ResNet50 2D-CNN, F1-score: 0.461) performed worse than models using image frames (Small 2D-CNN, F1-score: 0.674; ResNet50 2D-CNN, F1-score: 0.795) [40]. Optical flow presents the advantage of not being restricted to a certain camera angle. It can predict chews but not detect bites.

### 3.4. Active Appearance Model

AAM is a computer vision algorithm that uses the statistical model of an object’s shape and appearance. The model is optimized to detect differences between objects in consecutive video frames. The model parameter values are used for least square techniques (or spectral regression) to match the object’s appearance to a new image. The resulting data can be used for training a classifier.

Cadavid et al. (2012) was the only publication (one out of 13, 8%) that used AAM to distinguish between chewing and non-chewing facial actions, for which they achieved a precision of 93%, after cross-validation [33]. AAM can detect chews but not bites and generally it is not widely used.

### 3.5. Video Fluoroscopy

Video fluoroscopy is a moving X-ray examination of swallowing, which displays the bolus movement through the oropharyngeal anatomical structures. Physicians use video fluoroscopy to gain insights into the eating mechanisms and the problems concerning mastication (e.g., dysphagia, or choking) [59].

The only study (1 out of 13, 8%) to use video fluoroscopy was Kato et al. (2021) detected swallows in older adults in order to determine which foods are more appropriate for elderly people (accuracy not reported) [45]. Overall, video fluoroscopy can track swallow. However, the disadvantages of video fluoroscopy are its elevated costs and dimensions, which limit the use of this technology to the clinical setting. A summary of the advantages and disadvantages of all the methods reviewed is presented (Table 2).

## 4. Discussion

In this systematic review we determined the accuracy, advantages and disadvantages of the current video-based automated methods for eating behavior. The main methods found were facial landmarks, deep learning, optical flow, and active appearance model. These methods can detect bites, chews, food intake, and food liking. Facial landmarks can be used to count bites, chews, and food liking automatically (accuracy: 90%, 60%, and 25%, respectively). CNNs can detect bites and gesture intake detection (accuracy: 91%, 86%, respectively). AAM can be used to detect chewing (accuracy: 93%), and optical flow can be used to count chews (accuracy: 88%). To detect swallows, video fluoroscopy was the only method found; however, video fluoroscopy is not suited beyond a clinical setting. Facial landmarks are the most used method for detecting bites and chews automatically from video recordings of eating episodes.

To our knowledge, this is the first study that describes and gives an overview of video-based automated measures of eating behavior. Our study provides a comprehensive overview of the available methods for detecting eating behavior events automatically from video recordings. Currently, the manual annotation of eating episodes is a time-consuming and expensive task, which is prone to subjectivity and attentional lapses. Large prospective eating behavior studies are unfeasible using the manual annotation. Thus, there is a demand for the annotation process to be automated. To aid in realizing this, we provided a systematic overview of the methods to automate the annotation process. Facial landmarks can be used to count bites and chews by tracking facial and body motion during videos of eating episodes [35,36,37,42]. However, the camera’s distance, angle, occlusion, darkness and camera-lens focal length can limit their efficiency [60]. The reviewed publications used 2D facial landmarks methods only; however, such methods appear too stringent for the 3D real-world application due to their low performance in tracking facial motion from a side view. Facial landmarks predicted food liking [35,36]. However, these studies did not include emotion detection methods that can predict food liking [61,62]. A synergistic interaction between facial landmarks and emotion detection can enhance food liking predictions and consumer’s acceptance of new food products [63].

CNNs can be used to count bites [50,51,52,53]. CNNs can be used to model eating behavior gestures that include human hands and body (e.g., intake gestures consisting of fine cutting, loading the food, and leading the food to the mouth). CNNs are effective at recognizing differences between consecutive video frames (e.g., presence/absence of hand in proximity of the mouth). However, CNNs are not effective for tracking movement between consecutive video frames (e.g., jaw movement during chewing).

Optical flow can detect chewing [39]. When coupled with a facial detector, optical flow can track jaw movement during chewing. Optical flow is not restricted to the camera angle: it tracks motion regardless of the user’s position. To detect intake gestures, optical flow is not indicated. Rouast et al. (2020) showed that using frames as input (appearance analysis) outperformed motion streams as input (optical flow analysis) [40].

AAM can be used to distinguish between chewing and talking [33]. However, more recent methods for facial and object recognition are commonly preferred to automatically detect eating behavior events.

Video fluoroscopy is an accurate and non-invasive technology that can track swallows [45], however it is inappropriate and inaccessible for automating eating behavior analysis due to its costs and dimensions.

Several limitations may be recognized. First, there is a discrepancy in comparing accuracy and performance metrics, due to different study designs and data magnitude. Second, three conference papers were included, although their future full version may include more details or updates. Third, this study includes only methods that analyze video recordings, without considering the accuracy of other methods for automated eating behavior analysis (e.g., bone conduction microphone, algorithmic modeling from scales data, magnetic jaw displacement).

Future work should focus on addressing current issues to provide updated methods for the eating behavior field. The 3D facial landmarks should be applied to improve accuracy from a lateral camera angle. Furthermore, standard procedures should be established for camera angle, data extraction from video, reference facial landmarks, and algorithms to detect eating behavior events. Importantly, privacy concerns regarding face recognition should be addressed. To achieve automatic swallow detection, optical flow should be tested for tracking throat movement.

Only three publications [38,40,44] found in this study used free-living conditions: two with a 360 camera in the middle of the table, and one using a combination of video recordings from indoor, outdoor, and public spaces. For sensor-based detection, free-living is a valid setup to detect food intake [64]. In the future, video-based detection of eating events should extend to free-living conditions, possibly placing 360 cameras in different positions.

Video-based methods should consider how the awareness of being monitored affects social modeling of eating and eating intake (particularly for energy-dense foods) [65,66].

Future personalized nutrition recommender systems could be implemented by combining automatic eating behavior analysis with food and calories intake estimation [67]. Advancements should be implemented through open-source software, which can boost collaboration in the field. It is hoped that the developments of future methods will provide objective measures to conduct prospective studies and allow intervention strategies to decrease eating rate and, subsequently, overeating.

## 5. Conclusions

Based on this systematic review, the use of facial landmarks is the most promising method to detect eating behavior events automatically from video recordings because it is the only method that can detect both bites and chews. Improvements of this technology are needed to standardize procedures. CNNs can detect bites automatically and optical flow can detect chews automatically, but feasible method to detect swallows are currently lacking.

Ideally, future methods should detect bites, chews, and swallows from video recordings using inexpensive hardware with low computational requirements. Future methods should be implemented with open-source software to boost collaboration and development. The automated video analysis of eating episodes would improve eating behavior research and provide real-time feedback to the consumers to improve their weight status and health.

## Figures and Tables

**Table 1:** The results table presents the publications included in this review study. The following information is summarized from left to right: first author, year of publication, journal, methods, outcomes of the study, validation method, and precision (as reported in the paper).

First Author	Year	Journal	Methods	Study Setting	Device	Outcomes	Validity	Precision
Cadavid [33]	2012	Pers. Ubiquit. Comput.	Active Appearance Model (AAM) for face tracking, and spectral analysis on the temporal window of the model parameter values; binary support vector machine classifier for chewing events	Laboratory	Not reported; 37 videos at 24 fps; frame resolution: 640 × 480	Chewing detection	Manual annotation for chewing events	93% after cross-validation
Okamoto [34]	2014	IEEE International Conference on Multimedia and Expo Workshops	Mouth detector limited to the lower part of detected face; Chopstick detection using OpenCV Hough transform for straight lines	Laboratory	Smartphone Google Nexus 5 (2.3 GHz Quad Core, Android 4.4), inner camera; frontal view	Food intake estimation	N.A.	N.A.
Hantke [35]	2018	Proceedings of the 20th ACM International Conference on Multimodal Interaction	OpenFace facial landmarks extraction for tracking the mouth	Office room	Logitech HD Pro Webcam C920; 30 fps; resolution: 1280 × 720; frontal view	Food liking	Leave-One-Out Cross-Validation and SVM	Likability 0.583
Haider [36]	2018	Proceedings of the 20th ACM International Conference on Multimodal Interaction	OpenSMILE for facial landmarks extraction, coupled with OpenSMILE audio-feature extraction	Office room	Logitech HD Pro Webcam C920; 30 fps; resolution: 1280 × 720; frontal view	Food liking	Leave-One-Out Cross-Validation and active feature transformation	0.61
Konstantinidis [37]	2019	Computer Vision Systems	OpenPose for mouth and hands tracking; Deep Network (3 Conv + shortcut, 3 Conv + shortcut, 3 LSTM)	Laboratory	85 videos; Samsung digital camcorder; 1.5 m away from the subject; side view	Automatic bite detection	F-Score: 0.9173	0.9175
Qiu [38]	2019	IEEE 16th International Conference on Wearable and Implantable Body Sensor Networks	Mask R-CNN for 360-degree camera meal videos; Thresholds for assessing pixel intersection between hand-face and hand-food to infer eating events	Free-living (Indoor food sharing scenarios)	Samsung’s gear 360 camera; 1024 × 1024 pixels	Food intake estimation	N.A.	N.A.
Hossain [39]	2020	IEEE Access	Face detection with manual selection of the region of interest; CNN for bite/non bite classification; Optical flow for spatial chewing motion at every pixel	Laboratory	84 videos; SJCAM SJ4000 Action Camera; 1080p video at 30 fps; side view	Automatic count of bites and chews	Manual annotation with 3-button system and LabView software (custom-made)	Bites: 88.9% ± 7.4%; Chews: 88.64% ± 5.29%
Rouast and Adam [40]	2020	IEEE J. Biomed. Health Inform	CNN for hand-to-mouth movement in 360-degree meal videos	Free-living (Indoor group meal)	102 videos; 360 fly-4 K camera; 24 fps	Intake gesture detection	N.A.	F1-score: 0.858
Konstantinidis [41]	2020	Nutrients	OpenPose skeletal and mouth features extracted for training the RABiD algorithm. Two stream data: 2D coordinates and distances from mouth corners, and from upper body	Laboratory	Samsung digital camcorder; 1.5 m away from the subject; side view; resolution: 576p (720 × 576 pixels) at 25 fps	Meal duration and bite counts	Manual annotation (Noldus Observer XT)	F1-score: 0.948
Nour [42]	2021	Advances in Social Sciences Research Journal	Facial landmarks (dlib) for tracking jawline movement; OpenPose for 2D pose estimation	N.A.	N.A.	Real-time eating activity tracking	Manual annotation	N.A.
Park [43]	2020	Robotics and Autonomous System	Facial landmarks (dlib) for mouth-pose estimator; Algorithmic model for improving 3D estimation, location, and orientation of the mouth	Laboratory	Intel SR300 RGB-D camera	Robot active feeding assistance	Wrist-mounted camera	N.A.
Alshboul [44]	2021	Sensors	Time series data consisting of Euclidean distance between jaw/mouth landmarks and a reference facial landmark	Free-living (outdoors, indoors, and public spaces)	300 videos; Huawei Y7 Prime 2018 smartphone; 13 MP camera; resolution: 1080p at 30 fps; frontal view	Number of chews	Manual annotation (Intra-class correlation coefficient = slow: 0.96, normal: 0.94, fast: 0.91)	Avg Error ± SD: 5.42% ± 4.61 (slow chewing) 7.47% ± 6.85 (normal chewing) 9.84% ± 9.55 (fast chewing)
Kato [45]	2021	Gerodontology	Video fluoroscopy of swallowing for determining which foods are more appropriate for elderly people	Laboratory	N.A.	Association between masticatory movements and food texture in older adults	N.A.	N.A.

**Table 2:** Advantages and disadvantages of each method reviewed in this study.

Method	Advantages	Disadvantages	Open-Source
Facial Landmarks	Bite detection Chew detection	Camera angle and distance can impact performance Only tested in 2D No swallow detection	Yes
Deep neural networks	Bite prediction Human body detection	Not efficient for chewing prediction Hardware and software requirements No swallow detection	Yes
Optical flow	Chewing prediction No camera angle restrictions	No bite detection Motion analysis outperformed by appearance analysis No swallow detection	Yes
Active appearance model	Chewing detection	No bite detection Not widely used No swallow detection	Yes
Video fluoroscopy	Swallow tracking	Cost and dimensions	No

**Figure A1:**
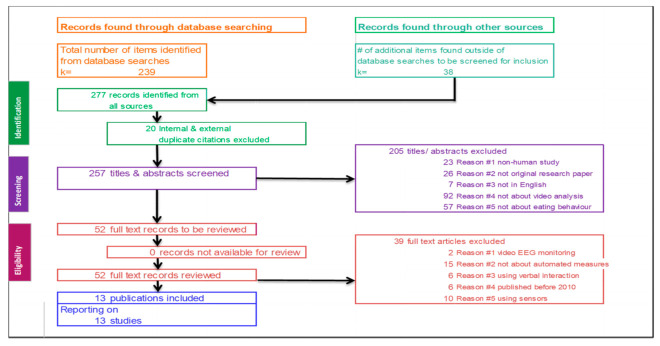
PRISMA flow diagram that depicts the identification, screening, and eligibility workflow for including the scientific records in this study.

## Data Availability

The data presented in this study are available on request from the corresponding author. The data are not publicly available due to ethical reasons.

## Appendix A

Search Queries

Scopus

(Only articles in English)

TITLE-ABS-KEY ((video AND eating AND behaviour OR behavior AND methodology OR chews OR bites OR meal OR machine AND learning OR computer AND vision OR automated OR analysis)) AND PUBYEAR > 2009

ScienceDirect

(With date range 2010–2022, all publication types)

video AND eating AND behaviour AND chew AND chewing AND bites AND meal AND machine learning AND computer vision AND human

PubMed

(With date range 2010–2022, all publication types, only human, only articles in English)

(((((video[Title/Abstract]) AND ((eating)[Title/Abstract])) AND ((behaviour[Title/Abstract] OR behavior)[Title/Abstract])) AND ((methodology)[Title/Abstract])) AND ((meal)[Title/Abstract] OR (chews[Title/Abstract]) AND (bites[Title/Abstract]) OR (meal[Title/Abstract]) OR (machine[Title/Abstract]) AND (learning[Title/Abstract]) OR (computer[Title/Abstract]) AND (vision[Title/Abstract]) OR (automated[Title/Abstract]) OR (analysis[Title/Abstract])) AND (computer[Title/Abstract] AND vision[Title/Abstract] OR automated[Title/Abstract] OR analysis)[Title/Abstract]))

Google Scholar

(With date range 2010–2022, all publication types, only articles in English)

human video eating behavior behaviour chew bites chewing meal machine learning computer vision automated analysis automatic methods methodology

PRISMA Flow Diagram
